# Dilated Cardiomyopathy: A Genetic Journey from Past to Future

**DOI:** 10.3390/ijms252111460

**Published:** 2024-10-25

**Authors:** Noah A. Newman, Michael A. Burke

**Affiliations:** 1 Department of Medicine, Emory University School of Medicine, Atlanta, GA 30322, USA; 2 Division of Cardiology, Emory University School of Medicine, Atlanta, GA 30322, USA

**Keywords:** genetics, dilated cardiomyopathy, arrhythmogenic cardiomyopathy, left ventricular non-compaction, heart failure, *TTN*, *LMNA*, polygenic risk score, peripartum cardiomyopathy, alcohol-induced cardiomyopathy

## Abstract

Dilated cardiomyopathy (DCM) is characterized by reduced systolic function and cardiac dilation. Cases without an identified secondary cause are classified as idiopathic dilated cardiomyopathy (IDC). Over the last 35 years, many cases of IDC have increasingly been recognized to be genetic in etiology with a core set of definitively causal genes in up to 40% of cases. While over 200 genes have been associated with DCM, the evidence supporting pathogenicity for most remains limited. Further, rapid advances in sequencing and bioinformatics have recently revealed a complex genetic spectrum ranging from monogenic to polygenic in DCM. These advances have also led to the discovery of causal and modifier genetic variants in secondary forms of DCM (e.g., alcohol-induced cardiomyopathy). Current guidelines recommend genetic counseling and screening, as well as endorsing a handful of genotype-specific therapies (e.g., device placement in *LMNA* cardiomyopathy). The future of genetics in DCM will likely involve polygenic risk scores, direct-to-consumer testing, and pharmacogenetics, requiring providers to have a thorough understanding of this rapidly developing field. Herein we outline three decades of genetics in DCM, summarize recent advances, and project possible future avenues for the field.

## 1. Introduction

The cardiomyopathies are a set of diseases with primary abnormalities in cardiac structure and function [1]. These disorders, which are typically grouped into morphologic subtypes, were associated with apparent familial transmission in many cases. This led to the hypothesis that the cardiomyopathies could have underlying genetic etiologies. Investigations over the last 35 years have confirmed a monogenic basis for these disorders. The first identified was hypertrophic cardiomyopathy (HCM), [2,3] which is now known to be a disease of the sarcomere, with >1500 variants identified in eight definitive sarcomere genes [4]. Similarly, causative variants for arrhythmogenic right ventricular cardiomyopathy (ARVC) have established ARVC as a disease of the desmosome [4], while channelopathies are predominantly caused by mutations in potassium and sodium channel genes [5]. Recently, additional genes have been identified that may contribute to these cardiomyopathies [6]. However, the evidence supporting their pathogenicity is less robust, frequently leading to diagnostic uncertainty in clinical testing [7,8,9].

Unlike these other cardiomyopathies, dilated cardiomyopathy (DCM) is unique in that it is genetically heterogeneous with a variety of culprit gene ontologies that lead to the common phenotype of a dilated and hypocontractile ventricle [1]. Further, the breathtaking pace of genetics discoveries, driven by disruptive technological advances in DNA sequencing and computing power, has led to a near constant evolution in our understanding and use of genetic information in patients with DCM. This review will discuss both the evolving genetic basis of DCM over the last three decades and the current and future impact of clinical genetic testing on the management of patients with DCM.

## 2. Epidemiology

DCM is a broad term that reflects the dilation and decreased systolic function of either or both ventricles [10]. Individuals with DCM typically develop heart failure (HF), and DCM is the most frequent underlying reason for heart transplantation. DCM is categorized into ischemic and non-ischemic etiologies, with nearly equal prevalence based on recent clinical trials in patients with reduced systolic function [11,12]. Many secondary causes of DCM exist including infections, drugs, toxins, nutrient deficiencies, and autoimmune diseases [13,14]. Historically, when a secondary cause could not be identified, DCM was classified as “idiopathic” (IDC). With progress in genetics, a substantial proportion of what used to be labeled IDC can now be called genetic. For the purposes of this review, we will focus on genetic causes of DCM.

Early and still widely cited epidemiologic data on DCM reported an incidence of ~6–8 per 100,000 population year [15,16,17] and a prevalence of ~1:2500 [17]. However, this is a substantial underestimate. When accounting for the prevalence of DCM among clinical trials and population cohorts it has been estimated that the true prevalence of DCM is closer to 1:250, or 0.4% [18]. This estimate has recently been confirmed using >39,000 patients imaged with cardiac MRI (CMR) in the UK Biobank. In this cohort, the population prevalence of DCM was found to be 1 in 220 (0.45%, 95% CI 0.39–0.53%), which encompasses the 1:250 estimate [19].

DCM carries a poor prognosis. In long-term studies of contemporary patient populations with non-ischemic DCM, roughly 50% experienced a composite of non-fatal life-threatening arrhythmia, unplanned cardiovascular hospitalization, or cardiovascular death over 12 years [20], and 17% experienced death, transplant, or ventricular assist device (VAD) implant over 8 years [21]. Prognosis has been noted to be worse in patients with pathogenic/likely pathogenic (P/LP) variants compared to those without an identified genetic contribution [22,23,24]. Notably, prognosis has improved when comparing patients diagnosed in the 1970s to those diagnosed in the 2010s [21], which may be in part attributed to increased utilization in guideline-directed medical therapy (GDMT), device therapies such as implantable cardioverter-defibrillators (ICD), and earlier patient identification [25].

## 3. Familial Clustering in Idiopathic DCM

Early studies suggested that 10–20% of patients with IDC have a family history [26]. However, a patient-reported family history is an insensitive marker for genetic disease. Among 109 probands with IDC, a reported family history was present in only 11%, while clinical screening with echocardiography identified DCM in the family members of 32% of probands, a nearly 3-fold difference [27]. Further, 44% of probands were found to have a disease-causing DCM variant, yet a family history was present in only 36% of these individuals. In a separate cohort of genotype-positive patients with DCM who were referred for VT ablation, a family history of DCM was present in only 27% and a family history of sudden death in 35% [28]. Indeed, in most large genetic studies of IDC, the prevalence of a reported family history is typically less than 50% even in those with putatively causal DCM gene mutations (i.e., in those with a clear heritable cause).

There are many reasons that explain the limited identification of familial disease in clinical practice (Table 1). An obvious reason is unrecognized disease in family members, a major problem in DCM as unsuspected but affected family members sometimes present with sudden death as the initial manifestation of disease [29,30]. This under-recognition of family members is partly due to the fact that virtually all causal DCM genetic mutations have (1) incomplete penetrance, meaning a percentage of individuals with a putatively causal DCM variant will never develop the phenotype; and (2) variable expressivity, meaning individuals with the same exact mutation will manifest different aspects of the disease phenotype and can have a vastly different disease severity. Additionally, the mutation could be a de novo variant in the proband, meaning there will be no history in siblings or older generations. This is referred to as sporadic DCM, another form of genetic DCM. Overall, while a family history increases the likelihood of finding a causal variant on genetic testing, the absence of a family history does not exclude the possibility of genetic disease. Further, a family history can be present in the absence of a pathogenic mutation because our understanding of DCM genetics remains incomplete.

The majority of genetic DCM occurs in adults, with a very variable age of onset, even within families. Consequently, the absence of the phenotype in a genotype-positive individual does not absolve them of future risk and underscores the need for lifelong screening as per guidelines [4]. Perhaps not surprisingly, when screening family members with cardiac imaging, the prevalence of familial DCM is higher due to identification of subclinical disease. The recent DCM precision medicine study estimated 30% of IDC to be familial. But, when utilizing an expanded definition that included either left ventricle (LV) chamber dilation or reduced LV ejection fraction (LVEF), which portend a 10-fold increased risk for progression to DCM [31], the prevalence of familial DCM was greater than 50% [32]. At the time of initial evaluation in seemingly asymptomatic relatives, up to 20% will be found to have overt DCM, with an age-dependent increase in prevalence [27,29,33]. Importantly, these individuals warrant proper treatment as per guidelines [34]. These facts underscore the guideline recommendations for both clinical and genetic screening in IDC [4,34].

## 4. Gene Ontologies in DCM

**Figure 1:**
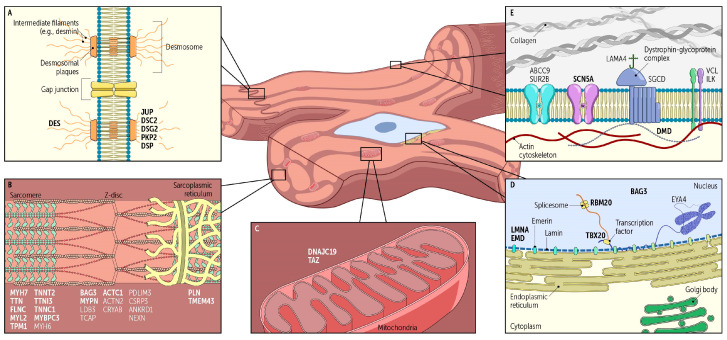
Visual illustration of the breadth of gene ontologies in dilated cardiomyopathy. Summarized are genes involved in (**A**) cell junctions, (**B**) the sarcomere, (**C**) mitochondria, (**D**) nuclear architecture and protein trafficking, and (**E**) cytoskeletal architecture (bold font—definitively pathogenic genes; standard font—putatively pathogenic genes).

Since the late 1990s, there have been tremendous advances in genetic sequencing allowing for even more nuanced appreciation of the influence of genetic variants on cardiomyopathy. DCM can result from a multiplicity of variants implicated in many different cellular processes. This includes genes involved in force generation, signaling, protein trafficking, electrolyte homeostasis, cellular architecture, energy regulation, and gene expression (Figure 1A–E and Table 2). Currently, mutations in over 250 genes have been associated with DCM. However, strong genetic evidence for the vast majority is lacking [7,8]. To define variant pathogenicity, the American College of Medical Genetics and Genomics (ACMG) and the Association for Molecular Pathology (AMP) published guidelines based on a diverse set of criteria. Scores are used to classify variants according to the following qualifiers: pathogenic (P), likely pathogenic (LP), variant of uncertain significance (VUS), likely benign (LB), or benign (B) [35].

### 4.1. Sarcomere Mutations in DCM

Similar to HCM, mutations in sarcomere genes are causal in DCM [37]. Definitive disease-causing variants in both disorders have been identified in cardiac actin (*ACTC1*), β-myosin heavy chain (*MYH7*), the troponins (*TNNT2*, *TNNI3*), and α-tropomyosin (*TPM1*), with putative DCM mutations also identified in cardiac troponin-c (*TNNC1*) and myosin binding protein C (*MYBPC3*) [1]. Sarcomere gene mutations underlie 30–40% of HCM and constitute nearly all P/LP variants on clinical genetic testing [127,128]. By contrast, sarcomere gene mutations are present in <10% of IDC cases [37,129,130,131,132].

Notably, detailed genetic and molecular analyses have shown that causal DCM and HCM variants in a given gene have opposing pathophysiologic effects. In a genome-wide association study (GWAS) of 1733 unrelated patients with HCM and 5521 patients with DCM, six loci were associated with HCM and three with DCM [133]. GWAS was also performed on LV phenotypic traits (e.g., LVEF, LV wall thickness, LV strain) in 19,260 participants who underwent CMR in the UK Biobank. This demonstrated strong associations between LV phenotypic traits, with opposing genetic effects in HCM and DCM. For instance, a positive genetic correlation was identified between HCM and LV wall thickness, while a positive genetic correlation was found between DCM and LV end diastolic volume, both disease hallmarks. Strikingly, all four measures of LV contractility assessed were positively correlated with HCM and negatively correlated with DCM. To identify additional genetic loci associated with HCM and DCM, the authors performed multi-trait analysis of GWAS (MTAG). MTAG improves the power of GWAS by identifying genetic loci that are associated with two correlated phenotypes (e.g., HCM and LVEF). This identified 10 additional loci for each HCM and DCM. Remarkably, 72.4% of these loci were significantly associated with both HCM and DCM, and of these, all but one were associated with an opposite direction of effect (i.e., if positively correlated with HCM, the risk allele was negatively associated with DCM and vice versa) [133].

This translates to in vivo models of sarcomere gene mutations. Distinct mutations in the *TNNC1* gene alter the calcium binding affinity of cardiac troponin-c [134], which in turn alters myofilament tension. Variants that increase calcium binding prolonged tension, producing hypercontractility, while those with lower calcium binding reduced myofilament tension and thereby contractility. This difference leads to differential activation of the nodal extracellular signal regulated kinase (ERK1/2) pathway. High myofilament tension increased ERK1/2 pathway signaling, while reduced tension blocks this activation. The result is concentric (HCM) or eccentric (DCM) cardiac hypertrophy, respectively. Thus, distinct cardiomyopathy mutations in the same gene can alter myofilament tension in very different ways, leading to marked differences in myocardial remodeling and, consequently, phenotype [134]. Collectively, these data prove that shared genetic pathways in HCM and DCM result in opposing pathophysiologic effects and demonstrate how genetics can provide a critical window into mechanistic understanding.

### 4.2. Nuclear and Cytoskeletal Architecture Mutations in DCM

Mutations in several structural genes also cause DCM, though the exact mechanisms remain unclear. These structural genes encode for key proteins in cytoskeletal and nuclear architecture. The most common of these structural genes, accounting for approximately 6% of all DCM cases, is *LMNA*, which encodes lamin A/C, a critical component of the nuclear lamina [135]. In humans, patients with *LMNA* variants have high disease penetrance (>90%) and a very high burden of electrophysiologic abnormalities including atrioventricular (AV) block, atrial fibrillation, and malignant ventricular arrhythmias (VAs) [136]. One third of cases of DCM with AV block are due to *LMNA* mutations [137], and nearly two thirds of individuals will have some degree of AV block within 7 years of a diagnosis of *LMNA* cardiomyopathy [138]. Risk factors that predict malignant VAs include (1) male sex; (2) non-missense mutation; (3) documented history of non-sustained VAs; and (4) LVEF < 45%. Defibrillator placement is reasonable in the presence of two or more of these risk factors [139]. These electrophysiologic complications are both progressive and highly prevalent in *LMNA* cardiomyopathy [138]. Consequently, when a cardiac implantable electronic device (CIED) is required for either a pacing or arrhythmia indication, placement of a dual chamber defibrillator is reasonable [139,140]. Hence, *LMNA* cardiomyopathy constitutes one of the few genotype-specific evidenced-based guideline recommendations in the cardiomyopathies.

A recently identified DCM gene, filamin C (*FLNC*) [74], binds to actin and is crucial for anchoring the cytoskeleton to the sarcomere and intercalated discs. It is required for sarcomere thin filament assembly [141,142,143]. Mutations in *FLNC* cause accumulation of Z-disc proteins, activate aberrant intracellular signaling, and alter cardiomyocyte calcium handling, which ultimately leads to reduced contractility [142,144]. *FLNC* mutations account for approximately 3% of DCM cases [74,131,145]. *FLNC* variants have also been associated with ACM [74] and HCM [77]. Interestingly, the type of variant seems to influence the phenotype: truncating variants almost exclusively cause DCM while missense variants predominate in those with HCM.

Structural genes implicated in DCM are often associated with syndromic forms of disease, most commonly with muscular dystrophy, secondary to their expression in both cardiac and skeletal muscle tissue. The archetypal syndromic cardiomyopathy is Duchenne muscular dystrophy, caused by mutations in the dystrophin (*DMD*) gene [81]. DCM is typically present in patients with Duchenne by their early 20 s. Mutations in *DMD* also cause Becker muscular dystrophy and X-linked DCM, a rare cardiac-only form of the disease [79]. Variants in *FLNC* have long been associated with myofibrillar myopathy, with cardiomyopathy only being identified recently. *LMNA* cardiomyopathy is associated with Emery-Dreifuss muscular dystrophy (EDMD) type 2, limb girdle muscular dystrophy, and congenital muscular dystrophy. Similarly, the inner nuclear envelope protein emerin, encoded by the *EMD* gene, is the cause of EDMD type 1, which is associated with cardiomyopathy in a majority of cases [146]. Mutations in the cytoskeletal protein desmin, encoded by the *DES* gene, cause DCM that frequently coexists with skeletal myopathy [82]. Skeletal muscle involvement has recently been found in the giant protein titin, though the clinical impact of these findings was unclear [147]. The presence of skeletal myopathy in other putative DCM genes as well as DCM being identified in other neuromuscular disorders pervade the literature. Importantly, when DCM is accompanied by neuromuscular disease, a P/LP variant is identified in >60% of cases on clinical genetic testing [129].

Mitochondrial genes also cause congenital forms of syndromic DCM. For example, Barth syndrome is a consequence of a rare X-linked mutation in the *TAZ* gene that encodes for tafazzin, which is responsible for altering mitochondrial membrane lipids [148]. This disease can be fatal in childhood and is further characterized by short stature and neutropenia [91]. Additionally, DCM with ataxia syndrome is an autosomal recessive disorder of the *DNAJC19* gene that encodes for the DnaJ heat shock protein family (Hsp40) member C19, an inner mitochondrial membrane protein. Predominantly occurring in a Hutterite population in Alberta, Canada, these patients are characterized by DCM and developmental delay with early mortality in childhood [149].

### 4.3. Protein Trafficking

The Bcl2-associated athanogene 3 (*BAG3*) gene encodes a multi-functional protein that is a structural component of the sarcomere and also functions to maintain normal cell function by regulating protein folding, apoptosis, autophagy, and mitochondrial function [150]. It is a very ancient protein found throughout the animal kingdom and has a close homolog in plants, suggesting it arose early in the development of multicellular life. Mutations have been identified in several DCM cohorts, with high penetrance [94,151,152]. Pathogenic mutations seem to reduce cellular BAG3 levels. Interestingly, BAG3 levels also decline in advanced HF, suggesting that reduced *BAG3* expression is a common pathway in both genetic and non-genetic forms of DCM, marking BAG3 as a particularly intriguing therapeutic target [151].

### 4.4. Titin

Despite the many disease-causing genes identified in the first two decades of the genetics era, the yield of clinical genetic testing for DCM remained frustratingly low (~10%). With the advent of massively parallel—so called “next generation”—sequencing (NGS) technology in the first decade of the new millennium, this would soon change with the identification of titin (*TTN*) as the commonest DCM-causing gene. Titin is, by far, the largest protein encoded by the human genome. A single titin protein spans half the length of the sarcomere to connect the Z-disc to the M-line [153]. It is essential for proper sarcomere assembly [154,155], and provides most of the passive tensile strength of the sarcomere [156,157]. Its incredibly large size made it cost-prohibitive to fully sequence in a large cohort of patients until the advent of NGS technology. Using NGS in a large, multi-center cohort of patients with DCM, researchers identified *TTN* mutations in over 21% of patients, doubling the yield of clinical genetic testing across other established DCM genes [56].

In this landmark study, *TTN* cosegregated in DCM families with a combined lod score of 11.1, unequivocally confirming the pathogenicity of *TTN* in DCM. However, putatively causal *TTN* variants were also identified in 3% of well-phenotyped controls without cardiac structural abnormalities, a prevalence roughly 20-fold greater than the estimated population prevalence of DCM. This created a vexing problem in establishing pathogenicity of novel variants on clinical genetic testing. An elegant molecular genetic analysis demonstrated that the majority of these pathogenic variants were located in exons that were not expressed in the final protein (e.g., the exon was spliced out of the transcript, yielding a normal TTN protein) [158]. In the remaining ~1% of ostensibly normal individuals harboring potentially pathogenic *TTN* variants in constitutively expressed exons, CMR demonstrated mild eccentric remodeling and mildly reduced contractility (i.e., subclinical disease) in most, revealing high penetrance but very variable expressivity for *TTN* in DCM [159]. Consequently, clinical genetic testing assesses not only the variant but whether that variant is in an exon that is part of the final mRNA transcript.

Importantly, the variants identified to be causal in *TTN* are truncating variants (*TTNtv*) that drastically alter titin structure by the removal of a large portion of the protein that variably contributes to distensibility, stretch-sensed signaling, and attachment to the M-line. When analyzed across a range of cohorts, *TTNtv* were identified in 11–15% of adult cases of sporadic DCM and 23–27% of familial DCM [56,158,159,160,161,162,163]. Assuming that up to 40% of DCM has a monogenic basis, then the approximate prevalence of currently actionable *TTNtv* in adult DCM is around 17–18%.

Non-truncating variants in *TTN* can also cause disease. A recent analysis identified non-canonical splice site variants as pathologic in an additional 1–2% of DCM cases [164], bringing the yield for *TTN* close to 20% in an unselected DCM cohort. Missense variants (those that cause a single amino acid change, leaving the protein intact) in *TTN* can also cause DCM as evidenced by several detailed familial studies [55,165,166]. Human induced pluripotent stem cells carrying putatively damaging *TTN* missense variants exhibit contractile dysfunction similar to *TTNtv*-expressing cells, further supporting causality of *TTN* missense variants [167]. Using in silico testing to predict pathogenicity suggests that TTN missense variants could be causal in up to an additional 5 to >20% of DCM cases [162,168,169]. However, due to the immense size of the *TTN* coding region, missense variants are extremely common, with an average of 23 per person [56,170]. When limiting to just rare variants with a minor allele frequency of at least <0.01, all individuals (i.e., not just those with DCM) carry ~1–2 rare *TTN* missense variants [56,168,170]. Importantly, in all but one study, *TTN* missense mutations are not enriched among DCM patients as compared to the general population [56,162,169,170]. Therefore, in the absence of detailed linkage studies in families of sufficient size, the majority of *TTN* missense variants currently remain uninterpretable (reported as VUS) on clinical genetic testing; this is a highly active area of research.

### 4.5. Gene Expression

The critical role played by *TTN* is supported by mutations in ribonucleic acid binding protein 20 (*RBM20*) gene [96], which encodes a nuclear protein that splices mRNA transcripts prior to their translation into protein. Interestingly, the molecular mechanism in the majority of *RBM20* variants appears to be the inability of an otherwise functional RBM20 protein to be transported into the nucleus [171]. Among many other genes, *RBM20* is necessary for the proper splicing of *TTN* mRNA [172]. In disease causing variants, a shift occurs in titin production from the N2B isoform to the larger and more compliant N2BA isoform, creating longer sarcomeres with altered Frank Sterling mechanics [173,174]. RBM20 also controls splicing of additional genes important in cardiomyocyte contraction, including those involved in Ca^2+^ regulation (*CAMK2D* and *CACNA1C*) [175], which may account for the notably high burden of VAs in these patients [176].

The T-box protein 20 (*TBX20*) gene, encoding a key developmental cardiac transcription factor, has long been associated with congenital heart defects [98]. While isolated cases in adult DCM have been reported, a recent analysis of nearly 7500 unrelated DCM probands identified substantial enrichment for *TBX20* truncating variants, making up 0.3% of all DCM cases [99]. Subsequent cosegregation analyses confirmed linkage with a lod score of 4.5. These individuals have a high prevalence of LV non-compaction (LVNC), and 34% also had concomitant congenital heart defects. This is the largest study of its kind and suggests other congenital defects may also constitute a small proportion of DCM cases.

## 5. Overlapping Cardiomyopathic Syndromes

### 5.1. Arrhythmogenic Cardiomyopathy

Arrhythmogenic right ventricular cardiomyopathy (ARVC) has been identified for decades [177] and is characterized by fatty infiltration and fibrosis of the right ventricle in association with HF and a particularly high burden of malignant VAs [178]. Early genetic studies defined ARVC as a disease of the cardiac desmosome with the identification of mutations in desmosomal genes plakoglobin (*JUP*), desmoplakin (*DSP*), plakophilin (*PKP2*), desmoglein 2 (*DSG2*), and desmocollin 2 (*DSC2*), and the non-desmosomal transmembrane protein 43 (*TMEM43*) gene [1]. Mutations in one of these genes may be found in ~60% of cases of ARVC [179,180].

With larger studies and improved imaging, particularly via CMR, it has become apparent that ARVC is not limited to the right ventricle. LV involvement is identified in 66–84% of cases of classic ARVC [181,182,183]. Up to 25% will have outright LV systolic dysfunction, with the causal gene having a large influence on this phenotypic trait (e.g., 0% LV systolic dysfunction with JUP and *TMEM43* mutations, but >50% with DSP) [182]. Conversely, classic ARVC has also been identified in several definitive DCM genes, namely phospholamban (~5% of those with a putative pathogenic mutation) [182], *LMNA* (~4%) [68], and *FLNC* (1–3%) [184,185].

**Figure 2:**
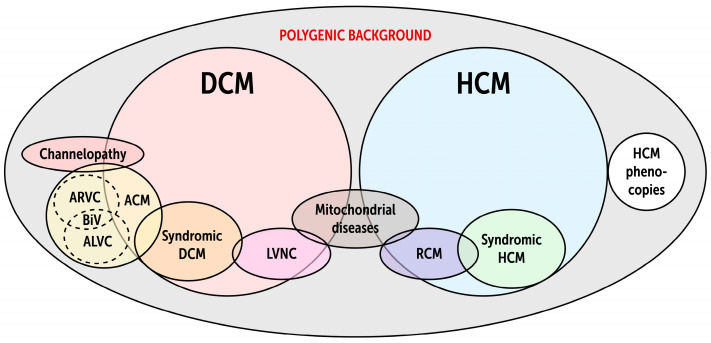
Summary of the genetic underpinnings of the various cardiomyopathy syndromes. Genes that produce channelopathies, ACM, LVNC, and mitochondrial disease can also produce a DCM phenotype. Similarly, genes implicated in RCM and mitochondrial disease can also produce an HCM phenotype. ACM arrhythmogenic cardiomyopathy, ARVC arrhythmogenic right ventricular cardiomyopathy, ALVC arrhythmogenic left ventricular cardiomyopathy, BiV biventricular, DCM dilated cardiomyopathy, HCM hypertrophic cardiomyopathy, LVNC left ventricular non-compaction, and RCM restrictive cardiomyopathy.

Improvements in imaging and the phenotyping of larger cohorts with mutations in specific genes has recently led to the discovery of ARVC-like features but with predominantly LV involvement, so-called ALVC. Because of this phenotypic heterogeneity, the consensus term now utilized in guidelines is “arrhythmogenic cardiomyopathy” (ACM), which encompasses ARVC and ALVC, and includes numerous known DCM genes that feature a high burden of arrhythmias in addition to the classic ARVC genes [34,186] (Figure 2). These include *LMNA*, *DES*, *FLNC*, *RBM20*, *BAG3,* and *TTN*. Additionally, multiple mutations in phospholamban (*PLN*), the molecular regulator of sarcoplasmic reticulum calcium cycling, cause DCM with a high burden of arrhythmias [121,122,123]. The altered flux of calcium between the sarcoplasmic reticulum and cytosol alters myofilament tension, leading to both reduced contractility [134] and electrical instability [122]. Mutations in the voltage-gated sodium channel α-subunit (*SCN5A*) can also cause DCM with VAs. *SCN5A* is a particularly promiscuous gene, with mutations that cause ACM, long-QT syndrome (type 3), Brugada syndrome, conduction delay, ectopic Purkinje foci, atrial fibrillation, and sinus node dysfunction via the varying effects of gain- or loss-of-function mutations that alter sodium current and membrane potential [187].

The desmosomal gene *DSP* is associated with a particularly unique form of ACM. DCM-causing mutations in *DSP* were originally described as part of Carvajal syndrome, an extremely rare autosomal recessive disease with DCM, palmoplantar keratoderma, and wooly hair [104]. With the contemporaneous discovery of the desmosome as the genetic basis of ARVC, rare autosomal dominant ARVC-causing mutations in *DSP* were soon identified [105]. However, subsequent work revealed that dominant mutations in *DSP* could also cause ALVC [188]. In a large natural history study of 107 patients with *DSP* cardiomyopathy, primary LV dysfunction was nearly four times as prevalent as RV-predominant disease and was associated with particularly poor outcomes [189]. Penetrance was also found to be much higher (nearly 60% by age 40) than what is typically seen with other desmosomal gene mutations. Interestingly, the disease is punctuated by episodic myocardial injury with troponin elevation mimicking acute myocarditis, a unique feature heretofore not definitively observed with other cardiomyopathy genes. This finding suggests that at least a subset of *DSP* cardiomyopathy might be responsive to immunosuppression, marking an active area of research that as yet is supported only by case reports [190].

### 5.2. Left Ventricular Non-Compaction Cardiomyopathy

Formation of LV trabeculations is a normal part of early embryologic development, with myocardial compaction occurring at later fetal stages [191]. LVNC defines a state where ventricular trabeculation exceeds normal parameters. This need not be found with aberrant cardiac function. Among healthy individuals, LVNC is common [192] and the prevalence is substantially higher in those who engage in vigorous physical activity [193]. Indeed, non-compaction is consistently identified in 1.5–8% of athletes [194,195] and in ~8% of healthy pregnant women [196]. Notably, excess LV trabeculation is not a static phenotypic trait, with both appearance and resolution occurring in various physiologic states [196,197]. The reason for this transience remains elusive.

Confounding our understanding of LVNC is a progressive change in diagnostic criteria [198]. Also, the prevalence of LVNC varies drastically by the imaging modality used: criteria for LVNC are met in approximately 1.3% of cases by echo, whereas this increases to 14.8% (a more than 11-fold difference) with CMR [199]. Among individuals with cardiomyopathy, LVNC can be a coexistent phenotype, most commonly with DCM [200], and also with HCM [201,202]. However, coexistent hypertrabeculation does not impact outcomes [198].

The best evidence supporting a genetic basis for LVNC comes from rare mutations in the mindbomb homolog 1 (*MIB1*) gene, a key component to the NOTCH signaling pathway, which plays a critical role in normal cardiac embryologic development [203]. Two distinct loss-of-function mutations were identified in two families with LVNC. However, subsequent modeling of these heterozygous mutations in mice failed to reproduce the phenotype [204]. This prompted whole exome sequencing that revealed co-inheritance of multiple modifier mutations with the *MIB1* variants in all affected individuals. These modifiers were required for development of LVNC in vivo, painting a complex genetic picture.

Other putative LVNC variants often display mixed phenotypes both within and across pedigrees. This variant-specific phenotypic heterogeneity most commonly coincides with DCM [165,205,206], but also occasionally with HCM, particularly apical HCM [207,208]. In large cohorts, the spectrum of putative pathogenic mutations largely resembles that of DCM, with variants in *TTN* being most common and multiple distinct gene ontologies identified, though HCM genes are also found [209,210,211,212]. Notably, in a cohort of 840 LVNC patients, a very small subset of mutations, particularly truncating variants in *MYH7*, were highly enriched in LVNC patients relative to controls or those with HCM and DCM, suggesting that a small subset of mutations within known cardiomyopathy genes either cause or act as modifiers to produce the LVNC phenotype [212].

These data demonstrate that the presence of LVNC is not necessarily a pathophysiologic state, and even when it is, there is substantial overlap with DCM, and to a lesser extent HCM, in the vast majority of cases. Importantly, LVEF is the primary driver of adverse events in LVNC and is a key metric to assess when considering therapy [213]. Because of this phenotypic pleiotropy, recent guidelines have dropped LVNC as a distinct cardiomyopathy, rather indicating that LVNC is a phenotypic variant of DCM and/or HCM, with the recommendation to manage patients according to guidelines specific for those disorders [214,215]. Whether a small subset of LVNC has a distinct underlying genetic architecture remains to be determined.

### 5.3. Restrictive Cardiomyopathy

Linkage analysis defined a mutation in *TNNI3* as the cause of restrictive cardiomyopathy (RCM) in a large family [216]. However, multiple family members met clinical criteria for HCM, and histologic analysis showed myocyte hypertrophy, fibrosis, and myofibrillar disarray consistent with HCM, a finding that has been observed in other patients with RCM [216,217]. Similarly, a mutation in the myopalladin (*MYPN*) gene was identified in RCM [64]. However, histologic analysis showed evidence for myofibrillar disarray, and other *MYPN* mutations have been associated with HCM [218] and DCM [65]. Additional mutations in *ACTC1* [217], *TNNT2* [217,219], and *MYL2* [220] have been associated with RCM, but phenotypic heterogeneity, including HCM and DCM, existed in affected first-degree relatives.

Restrictive physiology is an extreme phenotypic variant in HCM, being identified in 1.5–6% of cases, and portends a particularly poor prognosis [221,222,223]. While rare regardless of the subgroup, restrictive physiology appears to be more common with thin filament gene mutations in HCM [223]. Much like LVNC with DCM, these data suggest that RCM is indicative of a genetic predilection to restrictive physiology in the context of a different cardiomyopathy (HCM) rather than a distinct genetic entity.

## 6. Genetics in Secondary Forms of DCM

Approximately 25–30% of non-ischemic DCM is associated with an identifiable secondary cause [18]. Recent evidence has demonstrated that several of these secondary forms of DCM have at least a partial genetic basis. The commonest mutations identified in all populations are *TTNtvs*. Importantly, this suggests that these insults are non-genetic modifiers that may alter the penetrance and/or expressivity of *TTNtvs* and indicate that *TTNtvs* are a likely genetic predisposition to other secondary forms of non-ischemic DCM.

### 6.1. Peripartum Cardiomyopathy

Peripartum cardiomyopathy (PPCM) is defined as new onset DCM occurring between ~1 month prepartum and ~5 months postpartum, and has long been noted to have familial clustering in some cases [26,224]. Recent evidence from large multicenter cohorts has identified truncating variants in definitive DCM genes among 15% of unselected PPCM cases [225,226]. *TTNtvs* account for the majority, and constitute ~10% of all PPCM cases, a rate similar to that observed in sporadic DCM. Additional genes significantly enriched in PPCM include *FLNC*, *DSP*, and *BAG3* [226]. This subset of genes mirrors DCM and suggests that other genetic factors are likely to play a role (e.g., other DCM genes, missense variants, genetic modifiers). However, since PPCM is a rare disease, the power to detect even rarer variants will be limited.

### 6.2. Cardiotoxins

Similar findings have been noted in patients with chemotherapy-induced cardiomyopathy [227] and alcohol-induced cardiomyopathy [228]. In a diverse cohort of adult patients with DCM attributed to cancer therapies (> 90% anthracyclines), 13.4% had potentially pathogenic variants in known DCM genes [227]. This was nearly triple the rate found in unselected cancer patients from the Cancer Genome Atlas. Again, *TTNtvs* were the most common, accounting for 60% of all variants identified in adults, and being found in 8.1% of all adult patients with chemotherapy-induced DCM. Notably, in pediatric cancer, *TTNtvs* were less common, accounting for 5% of all cases. This is concordant with pediatric DCM data, where *TTNtvs* consistently make up a significantly lower percent of P/LP variants, particularly in younger children (6% in children < 13 years old) [229].

Alcohol-induced cardiomyopathy is classically defined when DCM is present in the absence of another cause in an individual that is consuming ≥80 g/day (approximately six each of: 12 oz beers that are 5% alcohol; 5 oz glasses of wine that are 12% alcohol; 1.5 oz shots of liquor that is 80 proof) for more than 5 years, a hard metric to quantify definitively. However, using this strict definition, 141 patients were identified who met criteria [228]. In these individuals, 13.5% were found to carry putatively causal variants in known DCM genes, again with *TTNtv* constituting the majority and being found in 10% of all individuals with alcohol-induced cardiomyopathy. Notably, over 40% of these individuals were found to have a family history of DCM.

After establishing the contribution of genetics to alcohol-induced cardiomyopathy, these authors sought to assess the role of moderate alcohol intake on DCM. Using a large and unselected DCM referral cohort, 15.5% were found to have “excess alcohol consumption” (defined by the authors as >24 g/day, similar to the upper limit of societal and government health guidelines across multiple countries) [228]. In a multi-variable analysis, neither the presence of a *TTNtv* nor excess alcohol consumption alone were associated with LVEF. However, in individuals with both a *TTNtv* and excess alcohol consumption, the LVEF was significantly and substantially (~10%; *p* < 0.01) lower than the cohort mean. This clever analysis identifies even moderate alcohol intake as a phenotypic modifier in DCM.

### 6.3. Acute Myocarditis

Acute myocarditis has confounded physicians for decades as the etiology typically remains elusive, even in the setting of a biopsy-confirmed histopathologic subset. While viruses have long been implicated, proving causality is extremely hard [230]. Recent evidence has shown there is a strong genetic predilection in at least a subset of cases. As noted above, there is a clear inflammatory subset among patients with *DSP* cardiomyopathy, a form of ACM [189]. In several cohorts of patients with biopsy-proven myocarditis, putatively causal DCM and ACM mutations are found in 8–33%, with *TTNtv* typically the commonest gene identified [231,232,233,234,235]. Notably, *DSP* mutations were identified in all cohorts.

Acute myocarditis presents with a very broad phenotypic spectrum from mild cases with normal LVEF to fulminant cases with severely reduced LVEF and cardiogenic shock. DCM-associated genes are more likely to be identified in myocarditis with reduced LVEF and shock [231,234,235]. By contrast in less severe cases and those with normal LVEF, fewer potentially pathogenic variants are identified, and the majority are in genes that cause ACM, in particular *DSP*. In nearly six years of follow-up, those with acute myocarditis and a pathogenic mutation were 3.1-fold less likely to have recovery of LVEF. Thus, identifying these individuals via genetic testing provides important prognostic information and could identify a cohort requiring closer long-term monitoring for maintenance of standard HF GDMT, though data supporting that approach remain limited.

## 7. The Natural History of DCM

With the dawn of the genetics era came great hope that genotyping would allow for gene-specific therapies for HF (i.e., pharmacogenomics). However, with the growth in our knowledge of cardiomyopathy genetics came the understanding that incomplete penetrance and variable disease expressivity were the norm in nearly all forms of genetic DCM. Within families, those carrying the same mutation can have starkly different clinical trajectories. Remarkably, this variability is even true in monozygotic twins [236]. This, coupled with the high cost of sequencing large cohorts of patients limited the ability to define gene-specific phenotypes that could in turn result in therapy tailored to the underlying cause.

The advent of NGS has permitted sequencing of large cohorts of patients with DCM at exponentially lower cost than was possible in the first two decades of the genomics era. Recently, this has led to several natural history studies of genetic DCM. Typically patients with genetic DCM are younger [23,237], have higher overall event rates [238], and, importantly, have an increased risk of progression to end-stage HF requiring advanced HF therapies including transplant or mechanical circulatory support (MCS) [23,237]. In the presence of a genetic etiology, patients also had higher likelihood of malignant VAs and are less likely to have reverse remodeling with standard HF treatments [23,24,237,239].

As more patients with genetic DCM have been identified, the power to detect differences between carriers of variants in specific genes has also grown. Several large studies in carriers of *TTNtv* have identified features unique to titin cardiomyopathy. While some studies show lower survival than IDC [158], the balance of evidence holds that *TTNtv* carriers have outcomes (death, transplant, MCS) that are similar to non-genetic DCM [23,56,160,240,241]. *TTNtv* carriers also have a higher incidence of VAs than non-genetic DCM [56,158] and may have poorer right ventricular function [158]. However, *TTNtv* seems particularly amenable to reverse remodeling, supporting the importance of GDMT in these individuals [23,242].

*LMNA* mutations produce the most malignant genetic DCM [243]. A hallmark feature of *LMNA* cardiomyopathy is a high burden of conduction system disease, atrial arrhythmias, and malignant VAs [138,243]. These are inexorably progressive, eventually develop in the majority of individuals, and guide management when considering CIED placement. *LMNA* cardiomyopathy also shows low rates of reverse remodeling [23,242] and very high rates of progression to end-stage HF [23,138,243]. Finally, *LMNA* mutations have high penetrance [243,244], which influences genetic counseling and makes cascade genetic testing particularly important.

Several other DCM genes have also been found to have disease-specific features. These influence prognosis and genetic counseling for patients and family members. *BAG3* mutations are highly penetrant (>80% by age 40), have high rates of malignant VAs, and a composite of death/transplant/MCS [152]. *FLNC* patients have a lower survival than those with *TTNtv* and have a high burden of malignant VAs [245,246]. Notably, this burden is identified even in those with only mildly reduced LVEF (36–49%) and the total burden is comparable to those with *LMNA* cardiomyopathy. This raises the consideration for defibrillator implantation with higher LVEF, though there is insufficient data for a specific recommendation. Sarcomere variants may have the highest penetrance among all DCM-causing genes [244], with penetrance of *MYH7* reaching nearly 90% by age 60 [247]. Over 1/3 of patients with *MYH7* variants will have LVNC, but notably, rates of malignant VAs are lower than most other forms of DCM and overall outcomes are comparable to *TTNtvs* [247]. Notably, the rate of adverse events is substantially higher in DCM-causing *MYH7* variants than in those causing HCM [248]. Understanding the natural history of distinct genetic causes is crucial for the future development of targeted therapies and is analogous to understanding the differences between different forms of cancer.

## 8. DCM: Beyond the Monogenic Hypothesis

The last 30 years have clearly defined a causal role for rare monogenic forms of DCM. Yet, putative pathogenic variants are only identified in ~40% of cases on genetic testing in unselected DCM cohorts. As noted, penetrance and expressivity are highly variable in DCM, and are influenced by genetic modifier variants [249], the epigenome, which is highly dynamic in DCM [250], and environmental factors, including other disease states, which are often associated with a worse prognosis [239]. With the rise of NGS and the sequencing of large cohorts, it is now apparent that rare putative disease-causing variants have a population prevalence that is much greater than that of overt or even subclinical DCM [19,251,252,253]. For instance, the penetrance of *TTNtvs* in the population may be as low as 10% [19]. It is important to note, however, that the penetrance of a putatively pathogenic variant in a family with the disease phenotype is much higher than those in an unselected population, a clear example of selection bias. This must be considered when providing genetic counseling.

These data may cast doubt on whether such mutations are truly causal. However, the sequencing and aggregation of very large population cohorts (e.g., gnomAD: https://gnomad.broadinstitute.org/ accessed on 19 April 2024) also permits burden testing—an aggregate statistical method used to identify phenotypically significant rare variants based on the difference between the prevalence of putative disease-causing variants in disease cohorts vs. controls. Burden testing has demonstrated a strong enrichment of most major DCM genes over the general population [7,132]. This cements pathogenicity when coupled with the robust genetic and functional data already published in these genes. In addition, by virtue of sequencing thousands of DCM patients and hundreds of thousands of individuals in the general population, burden testing essentially rules out the possibility of another as-yet-discovered gene making a large contribution to the monogenic basis of DCM. Extremely rare causal variants in new genes will continue to be found, but they will contribute only a very small percent to the etiology of IDC.

So, what of the 60% or more who have DCM without a definitive monogenic predisposition? Three GWASs and one exome-wide association study specifically focusing on idiopathic or sporadic DCM have been performed, identifying a collective 11 risk alleles [95,254,255,256]. Approximately half of the loci contain known DCM-causing genes, thereby establishing a link between common genetic variants and DCM, and identifying other genetic loci that are also involved. Using a discovery cohort of nearly 7000 individuals and eight identified common risk alleles for DCM, a polygenic risk score (PRS) was generated. A PRS is a cumulative score of single-nucleotide polymorphisms associated with a disease that can be used to determine disease associations and aid in prognosis. This PRS found a ~3-fold increased risk of DCM in individuals with eight vs. five risk alleles (the median of the population); similarly, there was a ~3-fold decrease in the risk of DCM in those with only one or two risk alleles [256].

Structural changes identified by imaging are a defining feature of DCM, are often perceptible before overt DCM is diagnosed, and are another powerful way to identify potential genetic loci [257]. A GWAS in over 36,000 patients without HF, DCM, or coronary disease who underwent CMR in the UK Biobank, identified 57 loci associated with some parameter of LV structure or function (45 of which had not previously been associated with DCM or cardiac imaging) [258]. A PRS for LV systolic function (highly correlated to DCM in the discovery cohort) was performed in the remaining participants in the UK Biobank (nearly 360,000 individuals) and found a strong association with incident DCM over nine years of follow-up. The polygenic background also influences the phenotype in *TTNtv* carriers. Per one SD increase in this PRS, LV end systolic volume increased by 7.2 mL and LVEF decreased by 2.6% [258].

**Figure 3:**
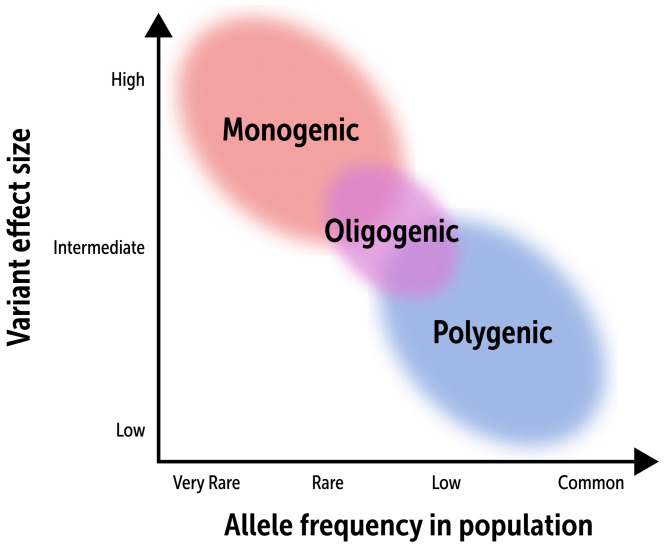
The relationship between variant effect size and its frequency in a population. Effect size refers to the magnitude of influence the variant has on a phenotype, whereas allele frequency describes the prevalence of the variant in a population. DCM is an example of a complex genetic disorder with a causal genetic basis ranging from monogenic, whereby very rare variants in specific genes have a marked effect on cardiac phenotype, to polygenic, where more prevalent minor variants, which individually have little effect on phenotype, can in aggregate result in the disease.

Collectively, these data demonstrate that genetic DCM is not simply a monogenic/Mendelian disorder, but rather a complex genetic disease that has both a monogenic and polygenic basis. The polygenic component of DCM also helps to explain the marked variance in penetrance and expressivity that is a hallmark of this disease (Figure 3). With continued refinement in larger and more diverse cohorts, PRS testing may become an important compliment to cascade genetic testing that can influence the potential penetrance and prognosis in family members with genetic DCM.

## 9. Using Genetics in the Management of DCM

The use of genetic testing is increasing rapidly in clinical practice. In addition, there has been a steady rise in direct-to-consumer genetic testing, with patients occasionally coming to clinic with genetic results in-hand. Consequently, understanding the utility and limitations of genetics and genetic testing is important for modern clinical practice.

### 9.1. Guideline-Based Recommendations

**Figure 4:**
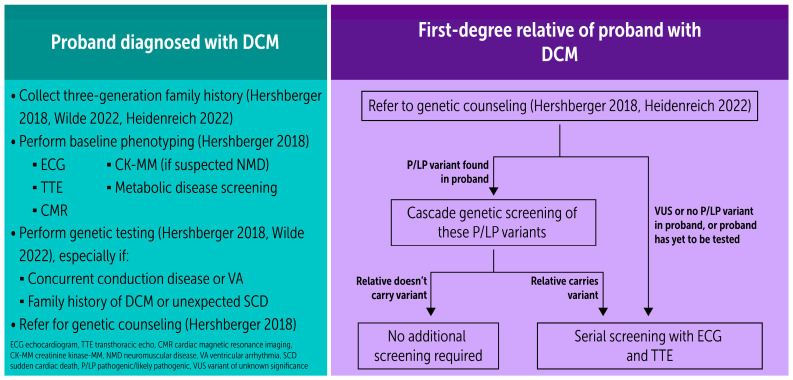
Summary of major guidelines recommendations to genetics screening in DCM. (**Left panel**) demonstrates steps provider should take for proband (index) patient. (**Right panel**) demonstrates algorithmic approach to first-degree relatives [4,34,259].

Numerous documents across multiple medical societies provide guidance on DCM (Figure 4). All guidelines recommend (1) collecting a three-generation family history [4,34,259]; (2) clinical screening for potential at-risk first-degree relatives; and (3) management at a center with expertise in genetic cardiomyopathies. The family history is often an afterthought in a busy clinical practice. A study comparing family histories taken by inpatient cardiology teams to those collected by genetic health care professionals demonstrated a 4-fold increase in the detection rate of a familial pattern by the latter group [260]. Further, reviewing ≥ three generations is useful in identifying the mode of inheritance and detecting variants with low penetrance [261]. These data support both the need for detailed family histories as well as the important role of the genetics counselor in cardiology practice.

There is also consensus across guidelines to offer cascade genetic screening and genetic counseling to first-degree relatives of patients with genetic DCM [4,34,259]. However, guidelines differ on which probands with IDC to refer. Guidelines and consensus documents endorsed by the ESC, HFSA, ACMG, HRS, and EHRA support genetic testing/counseling in all patients with IDC. By contrast, the recent AHA/ACC/HFSA HF guidelines only support testing of “select” patients with DCM without providing additional details. While controlled prospective studies have not shown a direct benefit of genetic testing, it informs prognosis, risk stratification and, in the case of *LMNA* cardiomyopathy, treatment. In a study of 4782 patients with suspected genetic cardiomyopathy or arrhythmia syndromes, genetic testing identified a putative pathogenic variant in 20% [262]. Of those gene-positive individuals, two thirds were variants that would alter prognosis and/or management. Importantly, had genetic testing only been offered to patients with a high suspicion of genetic disease, 14.4% of positive test results would have been missed [262]. Further, in first-degree relatives of probands with genetic DCM, cascade genetic testing was a more cost-effective approach to periodic clinical surveillance [263].

### 9.2. The Burden of VUSs

Commercial genetic testing panels include many genes with weak genetic evidence, and/or that have only been identified in a very small number of individuals. These weakly associated genes virtually always return VUSs because nearly all variants will be novel/private to that individual, and there simply is not enough data without detailed familial cosegregation studies to define pathogenicity [127,128,262,264]. For example, in 240 HCM patients who did not have a variant in one of the eight sarcomere genes, only one of 186 rare variants (0.5%) across 51 additional genes was reported as likely pathogenic, with 94.5% being VUSs [128]. Dealing with VUSs represents perhaps the single most challenging aspect of clinical genetics.

That said, using burden testing in DCM has shown that the prevalence of VUSs in many DCM genes far exceeds the population prevalence of rare variations in those genes, indicating that at least some of these VUSs are indeed pathogenic [7]. In silico tools are used to predict whether a particular variant will alter protein function. However, none are particularly sensitive. These in silico tools use machine learning and are trained using genome-wide data; consequently, they are not disease-specific, a major flaw that ignores disease-specific mechanisms or other evidence that may be known and aid in definitive variant prediction in a well characterized subset of disease-specific genes. With this in mind, a disease-specific prediction tool for inherited cardiomyopathies and arrhythmias has been developed (Cardioboost: https://www.cardiodb.org/cardioboost/ accessed on 1 June 2024) [265]. Cardioboost far outperforms other in silico tools. It reclassifies many variants that would otherwise be labeled as “indeterminate” and reduces the proportion of such variants by more than half as compared to genome-wide tools. We use Cardioboost to evaluate VUSs on clinical genetic testing in definitive DCM or arrhythmia genes. It is not meant as a stand-alone tool to change a VUS to a different classification but can aid in boosting a variant as part of the ACMG/AMP criteria (specifically as supporting evidence in criteria PP3 and BP4).

### 9.3. The Future of Genetic Testing

**Figure 5:**
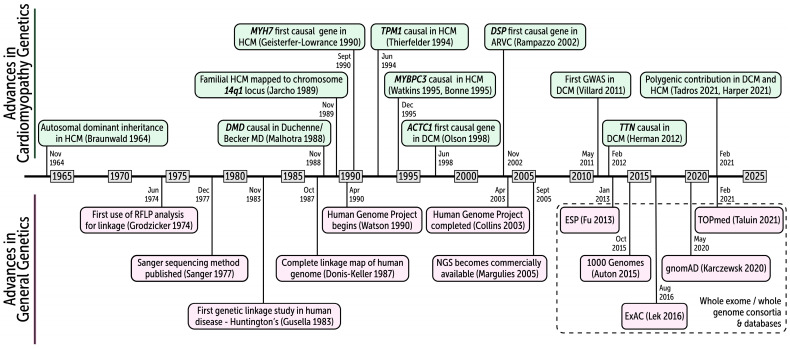
A 60-year history of advances in cardiomyopathy genetics. Our understanding of the genetic basis of the cardiomyopathies has paralleled major advances in genetics research tools. HCM hypertrophic cardiomyopathy; RFLP restriction fragment length polymorphism; MD muscular dystrophy; DCM dilated cardiomyopathy; ARVC arrhythmogenic right ventricular cardiomyopathy; NGS next-generation sequencing; GWAS genome-wide association study; ESP the NHLBI Exome Sequencing Project; ExAC exome aggregation consortium; gnomAD genome aggregation database consortium; TOPmed the NHLBI Trans-omics for precision medicine study. References in the figure include [2,3,36,43,53,54,56,81,95,105,133,266,267,268,269,270,271,272,273,274,275,276,277,278,279].

Genetics is one of the most rapidly progressing fields in all of cardiovascular medicine (Figure 5). The first 30 years can be considered the monogenic era: numerous genes were unequivocally defined to be causal in genetic DCM. This was greatly aided by the development of NGS which has both increased throughput and decreased costs by several orders of magnitude. While the future will likely bring more disease genes, burden testing strongly suggests that the majority of monogenic causes of genetic DCM have been identified, with the possible exception of the causal burden of *TTN* missense variants.

The field has now entered a new era of polygenic discovery. The future is sure to hold more sequencing of large cohorts with concomitant identification of more genetic loci. Hopefully, there will also be an increase in the diversity of population and disease cohorts, which remain dominated by those of European ancestry [280]. This will allow refinement of PRS testing, which will eventually aid in prognosis and genetic counseling. With continued decreases in sequencing costs, direct-to-consumer testing will also likely increase. This will pose challenges not only to patients struggling to interpret this information, but also to practitioners for how to counsel them. Investment is therefore needed in genetic counseling to address the growing proportion of individuals who will have access to genetic data.

Finally, we can anticipate the next era, which we hope will be dominated by therapeutic advances in DCM treatment guided by or generated as a result of genetic and molecular genetic discovery. Gene-specific medications are starting to become available [281], though none has yet proven effective. Gene editing therapies are also being investigated. For example, CRISPR-Cas9 technologies have been used to modify genetic DCM causing variants in mice and human stem cells with promising results [282,283]. In-human studies are far rarer—the first such clinical trial (NCT05836259) for genetic cardiomyopathy is currently ongoing that targets the *MYBPC3* gene implicated in HCM, but is still in the 1b phase [284]. Additional research is developing new viral vectors, also referred to as myotropic adeno-associated viral vectors (MyoAAVs), that are more specific to and effective at delivering therapeutic genes to cardiomyocytes [285].

In conclusion, the role of genetics continues to expand in both IDC and non-ischemic secondary causes of DCM and now encompasses fundamental aspects of the diagnosis, management, and prognostication of patients with DCM.

## Figures and Tables

**Table 1:** Challenges and solutions to identifying familial disease.

Challenge	Solution
Inadequate family history	-Take at least a three-generation family history (i.e., current, prior, and subsequent generations) -Clarify potentially vague cardiovascular events (e.g., heart attack) -Obtain family medical records for review
Incomplete penetrance and variable expressivity	-Evaluation by a cardiologist -Genetic testing of P/LP genes -Echocardiogram -ECG
De novo variant	-Cascade genetic testing in offspring

P/LP pathogenic/likely pathogenic, ECG electrocardiogram.

**Table 2:** Gene ontologies in dilated cardiomyopathy.

Gene	OMIM	Protein	Associated Phenotype(s) Other than DCM
Sarcomere			
*ACTC1* [36]	102540	Cardiac actin	HCM
*MYH7* [37,38]	160760	β-myosin heavy chain	HCM [3,39] and LVNC [40,41]
*TNNT2* [37]	191045	Troponin-T	HCM [42,43]
*TNNI3* [44,45]	191044	Troponin-I	HCM [46]
*TNNC1* [44]	191040	Troponin-C	HCM (putative) [47,48,49]
*TPM1* [50]	191010	ɑ-Tropomyosin	HCM [42,43]
*MYBPC3* [51,52]	600958	Myosin binding protein C	HCM [53,54]
*TTN* [55,56]	188840	Titin	ACM [57], Tibial Muscular Dystrophy [58], LGMD2J [59], Hereditary Myopathy with Early Respiratory Failure [60], and Salih Myopathy [61]
*MYL2* [62]	160781	Myosin light chain 2	HCM [63]
*MYPN* [64,65]	608517	Myopalladin	HCM (putative) [64]
Nuclear and cytoskeletal architecture			
*LMNA* [66,67]	150330	Lamin A/C	ACM [68], EDMD type 2 [69], LGMD1B [70], and Congenital Muscular Dystrophy [71]
*LEM2*	616312	LEM domain-containing protein 2 (LEMD2)	ACM [72,73]
*FLNC* [74]	102565	Filamin C	ACM [74,75], HCM (putative) [76,77], and MFM [78]
*DMD* [79,80]	300377	Dystrophin	Duchenne muscular dystrophy and Becker muscular dystrophy [81]
*EMD* [82]	300384	Emerin	EDMD type 1 [83]
*DES* [84,85,86,87]	125660	Desmin	LVNC [88,89], RCM [90], ACM and MFM [84,86]
Mitochondrial			
*TAZ* [91]	300394	Taffazin	Barth syndrome [91]
*DNAJC19* [92,93]	608977	DnaJ heat shock protein family (Hsp40) member C19	Dilated cardiomyopathy with ataxia syndrome [92,93]
Protein trafficking			
*BAG3* [94,95]	603883	Bcl2-associated athanogene 3	ACM, MFM
Gene expression			
*RBM20* [96]	613171	Ribonucleic acid binding protein 20	ACM [97]
*TBX20* [98]	606061	T-box protein 20	Congenital heart defects [98], LVNC [99]
Desmosomal proteins			
*JUP* [100]	173325	Plakoglobin	ARVC/ACM and Naxos syndrome [101,102,103]
*DSP* [104]	125647	Desmoplakin	ARVC/ACM [105] and Carvajal syndrome [104,106]
*PKP2* [107]	602861	Plakophilin	ARVC/ACM [108,109,110]
*DSG2* [107]	125671	Desmoglein 2	ARVC/ACM [111,112,113]
*DSC2* [100,107]	125645	Desmocollin 2	ARVC/ACM [114,115,116]
Membrane proteins			
*TMEM43* [117]	612048	Transmembrane protein 43	ARVC/ACM [117,118,119]
*ILK*	602366	Integrin-linked kinase	ARVC/ACM [120]
Sarcoplasmic reticulum			
*PLN* [121,122,123]	172405	Phospholamban	
Channels			
*SCN5A* [124,125,126]	600163	Voltage-gated sodium channel α-subunit	ACM, long-QT syndrome (type 3), Brugada syndrome, conduction delay, ectopic Purkinje foci, sinus node dysfunction [124], and atrial fibrillation [125]

HCM hypertrophic cardiomyopathy, LVNC left ventricular non-compaction, ACM arrhythmogenic cardiomyopathy, ARVC arrhythmogenic right ventricular cardiomyopathy, LGMD limb-girdle muscular dystrophy, EDMD Emery-Dreifuss muscular dystrophy, MFM myofibrillar myopathy.

## Data Availability

No new data were created or analyzed in this study. Data sharing is not applicable to this article.
